# Effects of parental exposure to glyphosate-based herbicides on embryonic development and oxidative status: a long-term experiment in a bird model

**DOI:** 10.1038/s41598-020-63365-1

**Published:** 2020-04-14

**Authors:** Suvi Ruuskanen, Miia J. Rainio, Maiju Uusitalo, Kari Saikkonen, Marjo Helander

**Affiliations:** 1 Department of Biology, University of Turku, Vesilinnantie 5, 20500 Turku, Finland; 2 Biodiversity Unit, University of Turku, Vesilinnantie 5, 20500 Turku, Finland

**Keywords:** Developmental biology, Ecology, Physiology, Zoology, Environmental sciences

## Abstract

Controversial glyphosate-based herbicides (GBHs) are the most frequently used herbicides globally. GBH residues are detected in soil, water, crops, and food products, potentially exposing non-target organisms to health risks; these organisms include wildlife, livestock, and humans. However, the potential for GBH-related parental effects are poorly understood. In the case of birds, GBHs may be transferred directly from mothers to eggs, or they may indirectly influence offspring performance by altered maternal resource allocation to eggs. We experimentally exposed a parental generation of Japanese quails (*Coturnix japonica*) to GBHs (200 mg/kg feed) or respective controls. Glyphosate residues were found in eggs (ca 0.76 kg/mg). Embryonic development tended to be poorer in the eggs of GBH-exposed parents (76% of eggs showed normal development) compared to control parents (89% normal eggs). Embryonic brain tissue from GBH-exposed parents tended to express more lipid damage (20% higher), yet other biomarkers showed no apparent differences. We detected no differences in egg quality (egg, yolk, or shell mass, egg hormone concentration) across the treatment groups. Given this is the first long-term study testing parental effects of GBHs with birds, more studies are needed characterizing GBH-associated changes in maternal allocation and for example epigenetic programming.

## Introduction

Glyphosate (N-[Phosphonomethyl]glycine)-based herbicides (GBHs) are the most frequently used herbicides globally and also one of the most controversial agrochemicals^1^. Evidence is accumulating with regard to the potentially negative effects of glyphosate on the development, phenotype, and fitness of most non-target animal taxa from invertebrates to vertebrates, yet, exposure levels (natural exposure load vs. levels used in experimental studies) need to be carefully accounted for^2–4^. Non-target organisms are commonly exposed to GBH residues in the food chain because residues can persist in soil, water, and plants^5,6^. In particular, the estimated amount of glyphosate introduced into the food chain through genetically modified, i.e. glyphosate tolerant crops (such as soybeans) add up to several thousands of metric tonnes yearly^7^. Consequently, different regulatory authorities heatedly debate the effects of GBH in our ecosystems.

Organisms in early developmental stages are generally more susceptible to external stress compared to adults. In the case of environmental toxins, this may be related to disturbed ontogeny or undeveloped detoxification metabolism in juveniles^8^. In aquatic animals, embryos can be directly exposed to GBHs via the surrounding water. Glyphosate and commercial products (e.g. RoundUp) made with glyphosate have been repeatedly reported to cause embryo mortality and deformations in fish (zebrafish 10uM to 1 mM pure glyphosate^9^, 10 mg/L RoundUp or pure glyphosate^10^), and aquatic amphibians (Xenopus 0.3–1.3 mg/L RoundUp^11^, 500 pg/egg pure glyphosate^12^). In contrast, mammal and bird embryos and fetuses are exposed to glyphosate residues only via maternal transfer of the chemicals, which may result in malformations, altered sex ratios, and low sperm quality in rodent models (doses: 500 mg/kg RoundUp^13^, 5 g/L pure glyphosate^14^, 50–450 mg/kg RoundUp^15^). Such effects are referred to as (transmissive) maternal effects sensu^16^. Furthermore, recent studies suggest that effects of GBHs on the next generation can be mediated via epigenetic paternal effects, for example via alterations of paternal sperm(^17^, parental generation, a dose of 25 mg/kg BW pure glyphosate in rats^18^).

However, the true maternal and paternal effects of GBH are poorly understood because the majority of the studies are involving direct embryo manipulations with high doses of GBHs. The authors of future studies should take into account that GBHs may influence the quality of the resources allocated to eggs/embryos and thus offspring development, phenotype, and fitness *indirectly*. Prenatal environmental conditions, and for example hormonal signals from the mother are known to have crucial importance for offspring development and even lasting effects into adulthood^19–21^.

In this study we used birds as a model to study the parental and developmental effects of GBHs. Birds are highly underrepresented in studies testing the adverse effects of GBH residues on non-target taxa^2^, although they have recently been suggested as a key group for biomonitoring with regard to the effects of GBHs^22^. The importance of poultry in food production also calls for more attention on residues and the effects of GBHs in birds. In the two available studies of poultry and GBH-related maternal effects, a direct injection of a relatively high concentration of RoundUp (10 mg/kg glyphosate) in eggs was found to decrease hatchability, induce oxidative stress and cause damage to lipids in the exposed chicks, as compared to the control group^23,24^, potentially via the disruption of retinoid acid signaling^12^.

To understand the potential for GBH-induced parental effects, we studied parental exposure of GBHs on embryo development and key physiological biomarkers—embryonic brain oxidative status in a bird model. To our knowledge, this is the first long-term study on parental effects of GBHs in bird taxa. Oxidative stress refers to the imbalance between reactive oxygen species (ROS) and antioxidants: If antioxidants are not able to neutralize ROS, oxidative damage to cell components (proteins, lipids, and DNA) will occur, which then has negative consequences on cell functions^25^. GBHs have been previously found to induce oxidative stress and damage in a variety of organisms and tissues, including embryos reviewed in^2^. We quantified glyphosate residues in eggs, but also maternal allocation to eggs (egg, yolk, and shell mass and yolk thyroid hormone concentration) to account for potential indirect GBH effects. Prenatal thyroid hormones (THs) (thyroxine, T4 and triiodothyronine, T3) play a key role in coordinating embryo development^20,26^, especially brain development^27^. Embryo THs have been reported to vary with maternal GBH exposure in rats (dose: RoundUp 5 and 50 mg/kg/day^28^), but generally the effects of GBHs on THs are poorly understood. Japanese quails were selected as the model species because the results can be applied to both wild birds feeding on GBH-contaminated food in the field and to poultry farming. We experimentally exposed parental bird generation to GBHs (ca 200 mg/kg feed) or respective controls from 10 days of age to 12 months. The egg samples were collected at 4 and 12 months to examine the potential cumulative effects of long-term exposure. We measured the potential effects on (1) on egg quality (egg, yolk, and shell mass as well as egg thyroid hormones); (2) on embryo development; and (3) embryo oxidative stress and damage.

## Results

We detected 0.76 mg/kg (S.D. ± 0.16) of glyphosate residue in eggs see also^29^, which is above the levels reported in the previous literature^30^. Egg mass from GBH and control parents did not differ after 4 or 12 months of exposure (treatment F_1, 17,1_ = 0.12, p = 0.73, treatment*exposure duration F_1, 270_ = 0.02, p = 0.89, Table 1) but was generally larger at 12 months of age (duration: F_1, 271_ = 8.8, p = 0.003). No differences between GBH exposed and control females in yolk mass, shell mass, or egg T3 and T4 concentrations were detected (Table 1, Suppl Fig. 1).

**Table 1:** Quality of the eggs (egg, yolk, and shell mass; thyroid hormone concentrations: T3 = triiodothyronine, T4 = thyroxine, average ± SD) from GBH (glyphosate based herbicide)-exposed and control females. The egg mass was averaged over all eggs (4 and 12 months of exposure). The other parameters were measured after 4 months of exposure. See text for power analysis.

Treatment	GBH	N (GBH)	Control	N (Control)	t_df_	*p*
Egg mass (g)	10.4 (0.8)	142	10.7 (0.9)	155	see text	
Yolk mass (g)	3.045 (0.296)	12	3.216 (0.254)	12	1.52_22_	0.14
Shell mass (g)	1.186 (0.122)	12	1.156 (0.147)	12	0.53_22_	0.59
T4 (pg/mg)	6.56 (0.96)	12	6.00 (1.66)	11	1.05_21_	0.30
T3 (pg/mg)	5.80 (2.18)	11	4.60 (1.6)	11	1.45_20_	0.16

**Figure 1:**
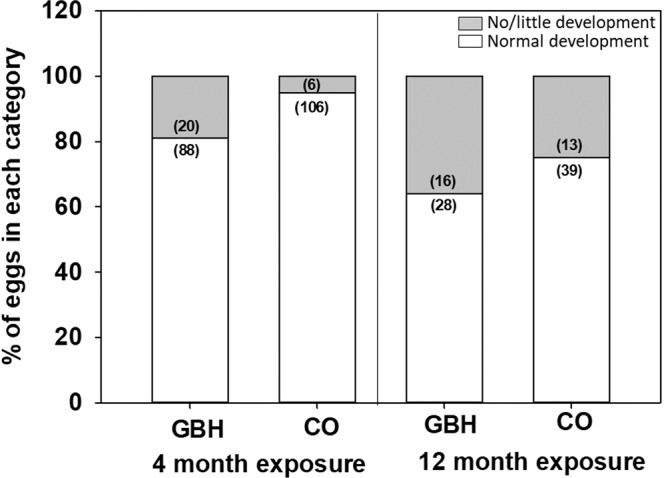
Embryonic status in relation to glyphosate-based herbicide exposure and duration of the exposure. GBH = glyphosate exposed, CO = controls. The bars are drawn separately for GBH and control eggs and after 4 and 12 months of exposure: we assessed 3-day-old embryos at 4 months and at 10-day-old embryos 12 months. Sample sizes are indicated in parentheses.

Embryo development was normal in 89% of control eggs, while 76% of GBH eggs had normally developed embryos when pooling data from 3-day and 10-day embryos. The lower percentage of normal development in GBH eggs tended to be statistically significant (treatment F_1, 22_ = 3.08, p = 0.09) and the trend was similar at both 4 and 12 months of parental exposure (treatment*duration F_1, 312_ = 0.6, p = 0.43, Fig. 1). The eggs with no or little development were distributed across pairs and for none of the pairs were all eggs classified as undeveloped. Brain mass did not differ between embryos from GBH-exposed and control parents (mean ± SD in mg; GBH: 67.1 ± 12.5, control 68.1 ± 15.5; F_1,31_ = 0.04, p = 0.84). Brain oxidative status at 12 months of parental exposure was measured from 19 control and 16 GBH embryos. We measured ca 20% higher lipid damage in the GBH embryos than controls. This difference tended to be statistically significant (F_1, 16.8_ = 3.2, p = 0.088, Table 2, Suppl Fig. 2), yet there were no differences in the activity of antioxidant enzymes (GST, GP or CAT) between the two groups (Table 2, Suppl Fig. 2).

**Table 2:** Average (±SD) of glutathione-S-transferase (GST), glutathione peroxidase (GP), catalase (CAT) activity, and damage to lipids (MDA) in 10-day-old Japanese quail embryos exposed to maternally-derived glyphosate-based herbicide (GBH) or unexposed embryos (control). Associate statistics from linear mixed models (LMMs) are reported below. See text for power analysis.

Treatment	GST (µmol/mg)	GP (nmol/min/mg)	CAT (µmol/min/mg)	MDA (µmol/mg)	N
GBH	0.0154 (0.004)	21.73 (4.91)	6.56 (2.96)	0.062 (0.016)	16
Control	0.0146 (0.003)	22.08 (4.28)	6.62 (2.29)	0.051 (0.018)	19
LMM Statistics	F_1, 15.8_ = 0.32 p = 0.57	F_1, 33_ = 0.05 p = 0.82	F_1, 33_ = 0.01 p = 0.92	F_1, 16.8_ = 3.2 p = 0.088	

## Discussion

Our results indicate that parental exposure to GBHs may lead to weak negative effects on embryo development and physiology. We detected no evidence for changes in egg quality (egg, yolk, shell mass, or egg hormone concentration), suggesting no indirect effects via the altered allocation of resources or hormones to eggs and embryos.

The tendency for poorer embryo development in eggs of GBH-exposed parents may be explained by GBH-related effects via either a paternal or maternal route, or both. We did not appear to observe cumulative effects of GBH exposure on embryo viability, as longer exposure (12 vs 4 months) did not lead to less viability. The eggs with no visible development (to the naked eye) could have been completely infertile or showing developmental arrest at an early stage. There are multiple potential mechanisms underlying such effects: For example, early embryos of fish showed developmental anomalies (disproportional head and body size) and increased heart rate (medeka, 100–500 mg/L RoundUp^31^, neurotoxic effects zebrafish, up to 50 mg/L RoundUp^32^) and Xenopus showed craniofacial deformities^12^. Poultry embryos and mice oocytes expressed increased oxidative stress (500 mM pure glyphosate^33^) and lower hatchability (10 mg/kg egg RoundUp^24^) in response to GBH exposure. Due to practical constraints we could only analyse embryo development until day 3 after 4 months of parental exposure while embryo day 10 was assessed after 12 months of parental exposure. The different timing of sampling can influence the fraction of embryos staged – after 10 days there are in general less viable embryos in both treatments. We do not currently know at what stage during the embryo development any potential negative effect of GBHs may occur. If it occurs mainly very early in the development, before our 3-day sampling point, then our two time-points should give comparable results. However, if potential negative effects of GBHs on embryo development mainly occurs after our 3-day sampling point, the 10-day sampling may reflect more appropriate sampling point. In the latter case, our study design cannot distinguish between short-term (4 months) and cumulative (12 months) of exposure as the sampling stage is confounded with sampling date. Due to rather limited sample sizes, the results need to be interpreted with care.

We selected embryonic development as a proxy of reproductive success because it is relevant for the poultry industry and for assessing GBH effects on population growth in wild species. Thus, our methodology could not distinguish between the alternative (paternal versus maternal) underlying mechanisms could have been completely infertile or show developmental arrest at an early stage. We hypothesized that potential infertility may result from GBH-related problems in semen quality or altered reproductive behavior in males as suggested previously in other vertebrates^2,34–36^. The fact that the measured egg quality parameters appeared not to be affected by GBHs suggests that any negative effects of GBHs on female folliculogenesis and ovary development, detected in previous studies^37–39^ are unlikely. Yet, a power analysis revealed that with the current effect sizes we have sufficient sample size to detect differences (with power 0.8) among the groups only for egg mass (estimated N = 94 per group compared to our 142–155 per group). However, for egg components, the required sample sizes (per group) were calculated as 32, 244, 75 and 58 for yolk, shell, T4 and T3, respectively, compared to our sample size of 12 per group, suggesting limited power.

We showed a tendency for GBH exposure to increase embryo brain lipid damage, while other biomarkers of oxidative stress appeared not be influenced. Potential effects on lipid damage could result from GBHs increasing reactive oxygen species (ROS) production^2^. Our results are in contrast to previous studies of poultry where embryos experimentally exposed to GBHs have shown large and significant teratogenic effects on early embryonic development, decreased antioxidant enzymes activities (GP, SOD, and CAT) and increased damage to lipids, liver tissue, and kidney tissue post-hatching^23,24^, However, our study design was different from previous studies, and the sample sizes in our study were relative small, so replication with larger sample sizes is needed to confirm our results. A power analysis revealed that with the current effect sizes, the sample sizes (per group) needed to find a statistically significant effect would have been 280, 2058, 22825 for GST, GP and CAT respectively, while only 32 for MDA, in contrast to our sample sizes of 16–19 per group. However, it must be noted that the abovementioned experimental studies using *in ovo* injections, researchers applied larger GBH doses compared to those in our experiment^12^ ca 50 mg/kg, i.e. >50x our study^24^, ca 10 mg/kg, >10x higher than residues in our study), which may explain the different responses. The embryonic brain can be particularly susceptible to ROS because the detoxification system via antioxidants is underdeveloped and polyunsaturated fatty acids are abundant in brain tissue. Indeed, the absence of an observed effect of GBHs on antioxidants in our experiment may be due to the incapability of the underdeveloped embryonic antioxidant system to respond to GBHs. Oxidative damage to lipids may have serious consequences on brain development^32^; it is also linked to aging and the onset of many diseases e.g.^40^ and GBHs have been shown to have neurotoxic effects on animals such as rodents^3,41^. It remains to be studied whether the small changes in embryo lipid damage can lead to measurable long-term effects in the offspring.

## Conclusions

Parental GBH exposure in a bird model appeared not to influence resource allocation to eggs, and had only weak effects on embryo development and physiology. However, to our knowledge, this is the first long-term study testing parental effects of GBHs with birds, and also taking into account the indirect effects via potential altered resource allocation. We only measured a limited number of biomarkers, and thus more studies are needed for characterizing GBH-associated changes in maternal allocation and epigenetic programming. Moreover, indications of negative effect in our study may not have been studied with sufficient statistical power. In a natural ecosystem, parental and transgenerational effects may lead to delayed and cascading impacts of agrochemicals; this may potentially explain why some non-target animal populations recover slowly after being exposed to environmental contamination. Thus, recurrent changes in wild populations or in production animals often remain unexplained and may be rarely linked to GBH exposure.

## Material and methods

We performed an experiment in which a parental generation of Japanese quails were fed with either GBH-contaminated food (N = 13 breeding pairs) or control food (N = 13 breeding pairs) from the age of 10 days to 12 months, and eggs and embryos from these pairs were collected. Details of the experimental design and parental exposures are described in^42^.

The GBH-exposed parents were fed organic food (Organic food for laying poultry, “Luonnon Punaheltta” Danish Agro, Denmark) with added commercial GBH (RoundUp Flex 480 g/l glyphosate, present as 588 g/l [43.8% w.w] of potassium salt of glyphosate, with surfactants alkylpolyglycoside (5% of weight) and nitrotryl (1% of weight) (AXGD42311 5/7/2017, Monsanto, 2002). The control parents were fed the same organic food in which water was added without GBH. A GBH product was selected over pure glyphosate to mimic the exposures in natural environments including exposure to adjuvants, as adjuvants may increase the toxicity of glyphosate^2,43^. However, with this experimental design we could not distinguish the potential effects of adjuvants themselves, or whether they altered the effects of the active ingredient, glyphosate. The concentration of glyphosate in the GBH food was aimed at ca 200 mg/kg food, which corresponds to a dose of 12–20 mg glyphosate/kg body mass/day in full-grown Japanese quails. Eason and Swanlon^44^ estimated that up to 350 mg/kg glyphosate could end up in grains when GBHs are spread on the fields before harvesting. However, the based on actual measurements, European Food Safety Authority (EFSA)^45^ estimated for poultry a maximum of 33.4 mg/kg glyphosate in feed (dry matter), corresponding to maximum intake of 2.28 mg/kg body mass daily. EFSA further reports a NOAEL (No Adverse Effects Level) of 100 mg/kg body mass/day for poultry^45^; therefore, our experiment tests a rather moderate concentration well below this threshold, yet above the estimated maximum residue by EFSA. Furthermore, a dose of 347 mg/kg did not negatively influence adult body mass in Japanese quails in a short-term experiment^44^. According to the manufacturer, acute toxicity (LC50) (via food) of RoundUp Flex is >4640 mg/kg food for mallards (*Anas platyrhynchos*) and bobwhite quail (*Colinus virginianus)*.

GBH food was prepared every week to avoid potential changes in concentration caused by degradation. Diluted RoundUp Flex was mixed with the organic food in a cement mill (Euro-Mix 125, Lescha, Germany). The food was air-dried and further crushed with a food crusher (Model ETM, Vercella Giuseppe, Italy) to a grain size suitable for the birds considering their age. The control food was prepared using a similar method, but only water was added to the food and a separate cement mill was used (ABM P135 L, Lescha, Germany). After crushing, the dry food was stored in closed containers at 20 °C in dry conditions. Separate equipment for food preparation and storage were used for GBH and control food to avoid contamination.

To verify the treatment levels in the parental generation, glyphosate concentration was measured in 6 batches of food and residue levels were measured in excreta (feces and urine combined) samples after 12 months of exposure. The average glyphosate concentration of 6 batches of food was 164 mg/kg (S.E. ± 55 mg/kg). The average glyphosate concentration in 3 pools of excreta samples (urine and fecal matter combined) was 199 mg/kg (S.E. ± 10.5 mg/kg). The control feed and control pools of excreta were free of glyphosate residues (<0.01 mg/kg).

### Egg mass

Parental generation was reared in same-sex groups for the first 12 weeks and thereafter in randomly allocated female-male pairs of the same treatment. Eggs were collected for measurements of egg mass when the birds were 4 and 12 months old. Eggs from each cage were collected eggs daily (quails generally lay one egg per day), marked individually, and weighed. A total of 221 and 96 eggs were collected at 4 and 12 months, respectively.

### Egg quality: Yolk and shell mass and thyroid hormones

Additional 24 unincubated eggs (N = 12 per group) were collected at 4 moths of exposure for egg component analyses. Eggs were thawed and the yolk and shell were separated and weighed (accuracy 1 mg). T3 and T4 were measured from yolk following previously published methods^46^ and were expressed as pg/mg yolk.

### Egg glyphosate residue analysis

When the birds were 10 months old, eggs from each cage were collected daily for 1 week, marked individually, and frozen at -20 °C for glyphosate residue analysis. Prior to analysis, randomly selected 5 eggs (originating from 5 different females) from both the GBH and control treatments were thawed and the shells carefully removed. To avoid contamination, all eggs were processed in a lab that was never in contact with glyphosate, using clean materials (gloves, petri dishes, tubes) for each egg. When removing content from the eggshell, the egg content was never in touch with the outer egg shell. The contents of 5 eggs from the control treatment were then pooled for glyphosate residue analysis. Pooling was done to reduce the costs of glyphosate analyses. The contents of 5 eggs from the GBH treatment were individually analysed for glyphosate residues.

### Development and tissue sampling

Embryo development was assessed after 4 and 12 months of parental exposure to GBHs. At 4 months, 108 GBH and 112 control eggs (see ‘egg mass’) were artificially incubated for 3 days at 36.8 °C and 55% humidity (Rcom Maru Max, Standard CT-190, Autoelex CO. LTD, South-Korea). Eggs were thereafter chilled and assessed for the presence of a normally developed embryo (coded 1) or no embryo/a very small embryo (coded 0) by naked eye. Note that by naked eye, one cannot distinguish between the unfertilized eggs and an embryo that died very early - yet the key idea was to study whether eggs developed to normal 3d-embryos or not to assess the effects of GBHs on development and reproduction.

After parental exposure for 12 months, 44 GBH and 52 control eggs were collected fresh and incubated for 10 days (i.e. 55% of the normal embryonic developmental period, 17 to 18 days) to assess general development (again, coded as 1 for normal development and 0 for no embryo/a very small embryo). The whole brain tissue of the embryo was dissected, snap frozen in liquid nitrogen and later stored at −80 °C for oxidative biomarker analysis.

The experiments were conducted under licenses from the Animal Experiment Board of the Administrative Agency of South Finland (ESAVI/7225/04.10.07/2017). All experiments were performed in accordance with relevant guidelines and regulations (Act on the Protection of Animals Used for Scientific or Educational Purposes, 497/2013).

### Brain mass and oxidative status biomarkers

We aimed to analyze 2 randomly selected embryo samples per breeding pair (N = 13 pairs/treatment). However, as not all females were producing eggs, or did not produce eggs with (viable) embryos, the final sample size was 19 control embryos (from 10 females) and 16 GBH embryos (from 10 females). Brain homogenates were used to measure oxidative status biomarkers, antioxidant enzymes glutathione-S-transferase (GST), glutathione peroxidases (GPx), catalase (CAT), and oxidative damage to lipids (malonaldehyde, MDA as a proxy, using TBARS assay). Whole brains were weighed (~0.1 mg) and homogenized (TissueLyser, Qiagen, Austin, USA) with 200–400 µl KF buffer (0.1 M K_2_HPO_4_ + 0.15 M KCl, pH 7.4). All biomarkers were measured in triplicate (intra-assay coefficient of variability [CV] < 15% in all cases) using an EnVision microplate reader (PerkinElmer, Finland) and calibrated to the protein concentration in the sample following^47^. The GPx-assay (Sigma CGP1) was adjusted from a cuvette to a 384-well plate. GPx was measured following kit instructions but instead of t-Bu-OOH, we used 2 mM H_2_O_2_, which is a substrate for GPx and CAT. To block CAT, 1 mM NaN_3_ was added and the pH was adjusted to 7.0 with the HCl in the buffer provided with the kit^48,49^. GST-assay (Sigma CS0410) was likewise adjusted from a 96- to a 384-well plate using our own reagents: Dulbecco’s Phosphate Buffered Saline–buffer (DPBS), 200 mM GSH (Sigma G4251), and 100 mM 1-Chloro-2,4-dinitrobenzene (CDNB) (Sigma C6396) in ethanol, see details in^50^. The CAT-assay (Sigma CAT100) was adjusted from a cuvette to a 96-well plate. We used a 0.3 mg/ml sample dilution, see details in^48,51^. The lipid peroxidation was analyzed using a 384-plate modification of a TBARS-assay following^52^.

### Statistical analysis

Egg mass was analyzed using linear mixed models (LMMs) with treatment (GBH or control), exposure duration (4 or 12 months) and their interaction as predictors, female mass as a covariate, and breeding pair ID as a random effect to control for non-independence of eggs from the same pair.

1 $${\rm{Egg}}\,{\rm{mass}}={\rm{Treatment}}\,\ast \,{\rm{Period}}+{\rm{Female}}\,{\rm{mass}}\,({\rm{covariate}})+{\rm{Pair}}\,{\rm{ID}}\,({\rm{random}})$$

Differences between the GBH and control groups in yolk and eggshell mass and egg thyroid hormone levels were analyzed with two-sample t-tests. The likelihood of embryo development was analyzed using generalized linear mixed models (binomial distribution, logit link):

2 $${\rm{Development}}={\rm{Treatment}}\,\ast \,{\rm{Period}}+{\rm{Female}}\,{\rm{mass}}\,({\rm{covariate}})+{\rm{Pair}}\,{\rm{ID}}\,({\rm{random}})$$

Embryo brain mass, GST, GP, CAT, and MDA were analyzed using LMMs as following:

3 $${\rm{Response}}={\rm{Treatment}}+{\rm{Pair}}\,{\rm{ID}}\,({\rm{random}})+{\rm{Assay}}\,{\rm{ID}}\,({\rm{random}})$$

The Kenward-Rogers method was used to estimate the degrees of freedom. Residuals of the models were visually inspected to confirm normality and heteroscedasticity. All statistical analyses were conducted with SAS Enterprise Guide 7.1. All data are available as Supplementary Data Files (1–3).

## Supplementary information

Supplementary Information.

Supplementary Information 2.

Supplementary Information 3.

## Data Availability

The datasets generated and analyzed during this study are available as Supplementary Data Files (1–3) and will be deposited in open repositories upon acceptance.
