# Supplementary material for: Effects of parental exposure to glyphosate-based herbicides on embryonic development and oxidative status: a long-term experiment in a bird model
Source: Sci Rep. 2020 Apr 14;10:6349. doi: 10.1038/s41598-020-63365-1 (PMC7156732; doi:10.1038/s41598-020-63365-1)
Supplement: Supplementary file 1 — Supplementary Information. [file 41598_2020_63365_MOESM1_ESM.docx]

Supplementary material to

Ruuskanen S, Rainio M, Uusitalo M, Saikkonen K, Helander M. Effects of parental exposure to glyphosate-based herbicides on embryonic development and oxidative status: a long-term experiment in a bird model.

1. Supplementary Fig 1 and Fig 2.
2. Datafiles 1-3
