# Supplementary material for: Effects of parental exposure to glyphosate-based herbicides on embryonic development and oxidative status: a long-term experiment in a bird model
Source: Sci Rep. 2020 Apr 14;10:6349. doi: 10.1038/s41598-020-63365-1 (PMC7156732; doi:10.1038/s41598-020-63365-1)

Supplementary Fig 1. Average (±SD) of egg parameters a) eggs mass, b) yolk mass, c) shell mass, d) egg thyroxine (T4) concentrations, and e) egg triiodothyronine (T3) concentration, GBH (glyphosate based herbicide)-exposed and control females. The egg mass was averaged over all eggs (4 and 12 months of exposure). The other parameters were measured after 4 months of exposure.

Supplementary Figure 2. Average (±SD) of a) glutathione-S-transferase (GST), b) glutathione peroxidase (GP), c) catalase (CAT) activity, and d) damage to lipids (MDA) in 10-day-old Japanese quail embryos exposed to maternally-derived glyphosate-based herbicide (GBH) or unexposed embryos (control). Sample sizes N(GBH) =16, N(control) = 19.


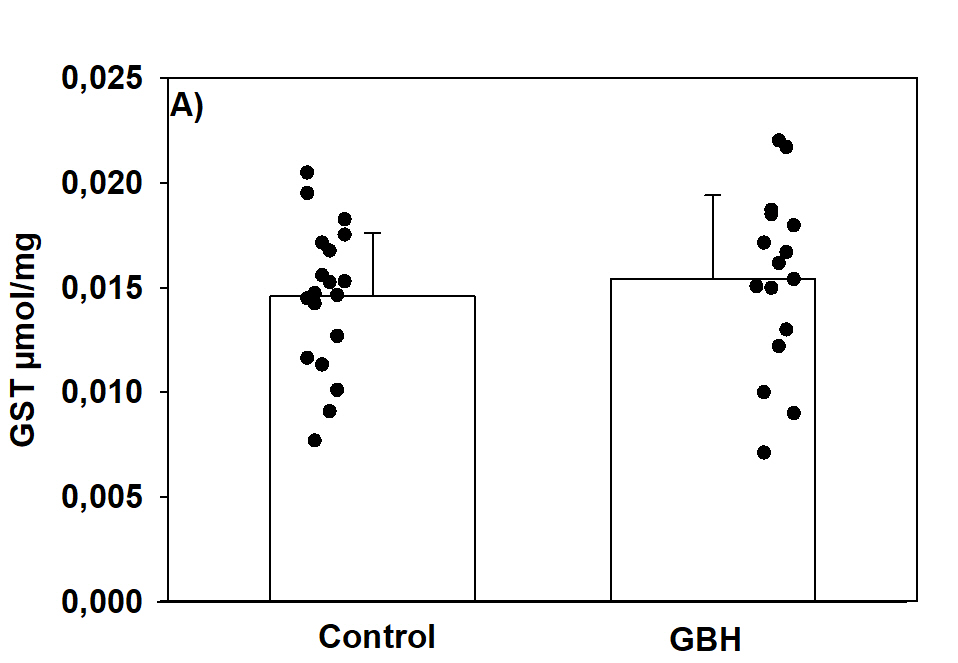

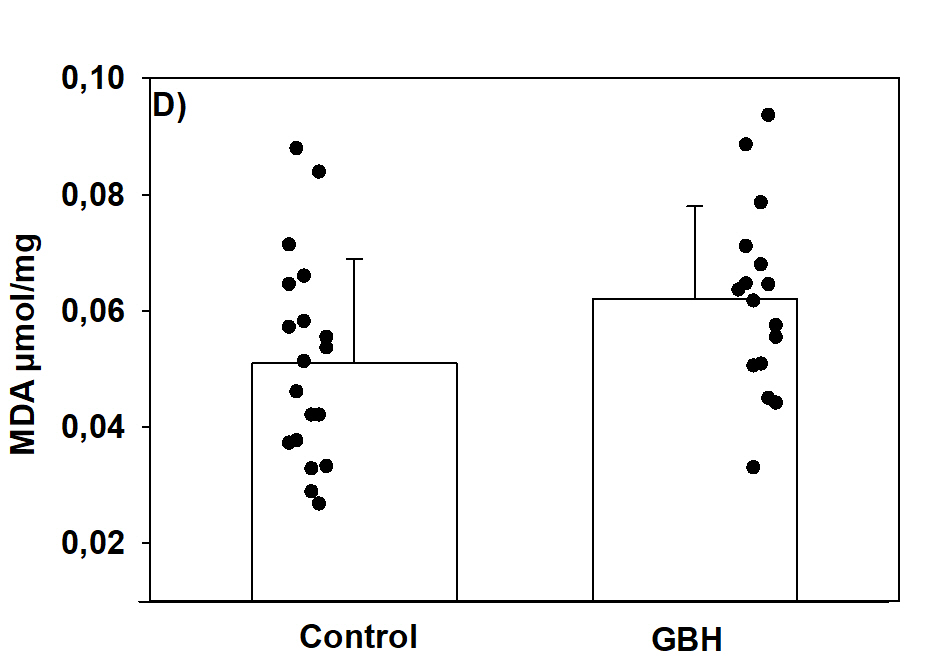

Supplement: Supplementary file 2 — Supplementary Information 2. [file 41598_2020_63365_MOESM2_ESM.docx]
